# Preparation, Characterization and Thermal Degradation of Polyimide (4-APS/BTDA)/SiO_2_ Composite Films

**DOI:** 10.3390/ijms13044860

**Published:** 2012-04-17

**Authors:** Mansor Bin Ahmad, Yadollah Gharayebi, Mohd. Sapuan Salit, Mohd. Zobir Hussein, Saeideh Ebrahimiasl, Arash Dehzangi

**Affiliations:** 1 Department of Chemistry, Universiti Putra Malaysia, 43400 UPM Serdang, Selangor, Malaysia; E-Mail: mzobir@science.upm.edu.my; 2 Advanced Materials and Nanotechnology Laboratory, Institute of Advanced Technology (ITMA), Universiti Putra Malaysia, 43400 UPM Serdang, Selangor, Malaysia; E-Mails: masoud.gharayebi@gmail.com (Y.G.); ebrahimi.saeideh@yahoo.com (S.E.); 3 Department of Chemistry, Islamic Azad University Behbahan Branch, University Street, Behbahan, 6361713198, Iran; 4 Department of Mechanical and Manufacturing Engineering, Universiti Putra Malaysia, 43400 UPM Serdang, Selangor, Malaysia; E-Mail: sapuan@eng.upm.edu.my; 5 Department of Physic, Universiti Putra Malaysia, 43400 Serdang, Selangor, Malaysia; E-Mail: dehzangi_ar@yahoo.com

**Keywords:** polyimide, silica, activation energy, thermogravimetric analysis

## Abstract

Polyimide/SiO_2_ composite films were prepared from tetraethoxysilane (TEOS) and poly(amic acid) (PAA) based on aromatic diamine (4-aminophenyl sulfone) (4-APS) and aromatic dianhydride (3,3,4,4-benzophenonetetracarboxylic dianhydride) (BTDA) via a sol-gel process in *N*-methyl-2-pyrrolidinone (NMP). The prepared polyimide/SiO_2_ composite films were characterized using X-ray diffraction (XRD), Fourier transform infrared spectroscopy (FTIR), scanning electron microscope (SEM) and thermogravimetric analysis (TGA). The FTIR results confirmed the synthesis of polyimide (4-APS/BTDA) and the formation of SiO_2_ particles in the polyimide matrix. Meanwhile, the SEM images showed that the SiO_2_ particles were well dispersed in the polyimide matrix. Thermal stability and kinetic parameters of the degradation processes for the prepared polyimide/SiO_2_ composite films were investigated using TGA in N_2_ atmosphere. The activation energy of the solid-state process was calculated using Flynn–Wall–Ozawa’s method without the knowledge of the reaction mechanism. The results indicated that thermal stability and the values of the calculated activation energies increased with the increase of the TEOS loading and the activation energy also varied with the percentage of weight loss for all compositions.

## 1. Introduction

Polyimides are a type of organic polymers that have been widely used in many applications at high temperatures, such as in aerospace, microelectronic industries [1,2], semiconductor and composites [3]. They demonstrate many advantages, which include excellent heat and chemical resistance [4], as well as outstanding combinations of thermal, mechanical and electrical insulating properties [5,6]. Thermal property is one of the most important properties for polymeric materials. Many researchers have studied the polyimides properties, and most of them have reported excellent thermal stability for polyimides [7]. Meng *et al*. (2007) have reported the thermal properties of a polyimide based on 2,6-bis(*p-*aminophenyl)-benzo[1,2-*d*;5,4-*d*]bisoxazole. The results showed excellent thermal stability, a 5% weight loss temperature (*T*_5%_) and glass transition temperatures (*T*_g_) at 572 °C and 283 °C in N_2_ respectively. Meanwhile, thermal stability and thermal degradation kinetics are significant to production and application [8]. In particular, thermogravimetric analysis (TGA) has been widely employed to investigate thermal degradation kinetic and thermal stability of polymers [9–13]. However, polyimides have many intrinsic weaknesses such as low thermal coefficient, poor corona-resistance property and comparatively high thermal expansivity, which can cause restrictions in some of their applications [14]. For enhancement and to obtain the desired improvements of polyimides many research activities have been carried out through using a mixture of inorganic in polymer matrices. The sol-gel process is an important method for the preparation of these hybrid materials, whereby both organic and inorganic elements are mixed at a molecular level and the prepared intimate mixing provides various properties which are different from those of the traditional composites [4].

In the present study, polyimide/SiO_2_ composite films were prepared from poly(amic acid) (PAA) based on aromatic diamine (4-Aminophenyl sulfone) (4-APS) and aromatic dianhydride (3,3′,4,4′-benzophenonetetracarboxylic dianhydride) (BTDA) with tetraethoxysilane (TEOS) as the SiO_2_ source via sol-gel process. The prepared composite films were characterized using by Fourier transform infrared (FTIR) spectroscopy, X-ray diffraction (XRD), scanning electron microscope (SEM) and thermogravimetric analysis (TGA). Thermal degradation and kinetic parameters, such as activation energy of thermal degradation processes were also investigated through dynamic thermogravimetric analysis at different heating rates.

## 2. Results and Discussion

### 2.1. Characterization of Polyimide/SiO_2_ Composite Films

#### 2.1.1. Chemical Analysis by FTIR Spectroscopy

**Figure 1:**
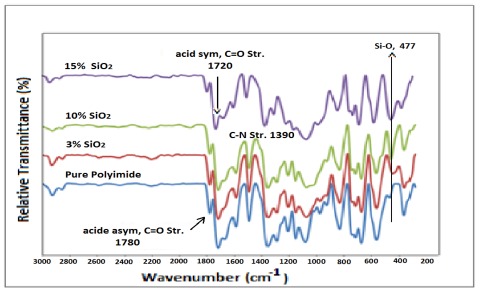
Representative Fourier transform infrared (FTIR) spectra of polyimide (4-APS-BTDA) and its composite films with various percentages of SiO_2_.

The FTIR spectra of the prepared polyimide/SiO_2_ composite films, with different contents of silica, are depicted in Figure 1. The characteristic absorption bands of the imide groups near 1780, 1720 and 1378 cm^−1^ were observed in the FTIR spectra of the prepared samples after thermal imidization of the poly(amic acid)/SiO_2_ precursor. Meanwhile, the characteristic absorption of the amide carbonyl at 1650 cm^−1^ did not appear in the spectra, indicating that the imidization reaction is complete [15]. The characteristic vibration bands of Si–O–Si hydrolyzed from silica were also observed at 477 cm^−1^ and near 1100 cm^−1^. As the content of SiO_2_ particles increased, the intensity of Si–O–Si band gradually became stronger in the FTIR spectra of the polyimide/SiO_2_ composite films [16].

#### 2.1.2. X-ray Diffraction Study of Polyimide/SiO_2_ Composite Films Structure

**Figure 2:**
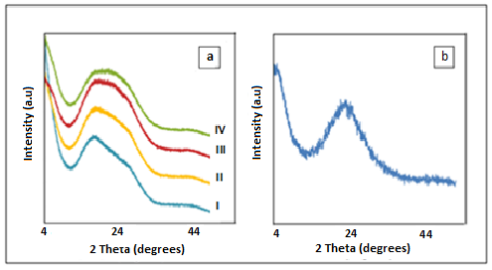
XRD patterns of (**a**) the polyimide/SiO_2_ composite films with (I–IV) 0, 10, 30 and 50 wt% TEOS respectively, and (**b**) SiO_2_ particles.

The prepared composite films were also characterized by XRD. Figure 2a shows the XRD patterns of the polyimide/SiO_2_ composite films with various contents of SiO_2_, prepared according to processing conditions in Section 3.3. Figure 2b shows the XRD pattern of prepared SiO_2_ particles in the same conditions but in the absence PAA where the average size of obtained particles was 610 nm. As is clearly seen in Figure 2a, there is a peak in the diffractogram of the polyimide (curve I) as the non-Gaussian distribution pattern that reveals a semi-crystalline structure polymer. This peak was also depicted in the all diffractograms of polyimide composite films. As the loading of TEOS increased in the PAA precursor (curves II–IV), the peaks shoulder after 2θ = 16 also heightened, suggesting that this could be due to the formation of SiO_2_ particles and increase of SiO_2_ particles content in polyimide matrix.

#### 2.1.3. Morphology of the Polyimide/SiO_2_ Composite Film

**Figure 3:**
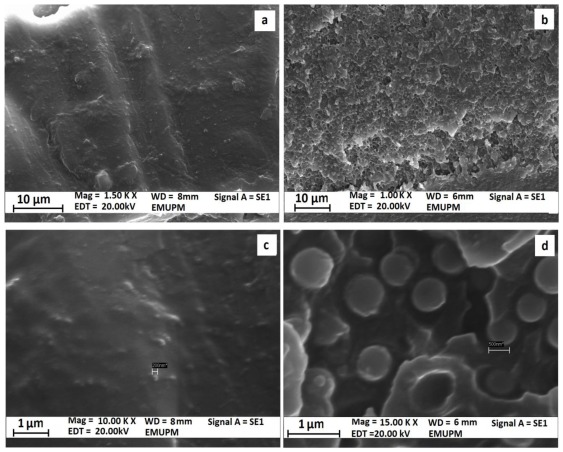
SEM photographs of the cross-section surfaces of polyimide/SiO_2_ composite films with various percentages of TEOS. (**a**) and (**c**) images: 10 wt% TEOS loading, (**b**) and (**d**) images: 50 wt% TEOS loading.

The SEM photographs of the cross-section surfaces of polyimide/SiO_2_ composite films in various percentages of TEOS as SiO_2_ sources are shown in Figure 3. The created SiO_2_ particles which are in white globular shapes have dispersed into the polymer matrix uniformly. The average size of the SiO_2_ particles in the composite films were estimated to be around 265, 374, 580 nm for the prepared composite films with 10, 30, 50 wt% of TEOS loading respectively. The sizes of the SiO_2_ particles for the polyimide composite films with various percentages of TEOS loading are also compared in Table 1. On the basis of the morphological observations, with the increase of the TEOS loading, the SiO_2_ particles size were increased which can be seen from the increase in the aggregation leaning of the SiO_2_ particles. The SEM images also revealed that with the increase of the TEOS loading, the dispersion of the SiO_2_ particles in the hybrid also became more uniform. The adhesion of the silica particles with the polyimide matrix is low as the particles seemed to have been completely debonded from the surrounding polyimide matrix, indicating a very poor interfacial adhesion between the particles and the matrix. The comparison of the SEM images also indicated that in higher contents of TEOS loading, the interfacial adhesion decreased. As is clearly seen, the distribution and dispersion of the SiO_2_ particles within the polyimide matrix are relatively uniform and this factor can be effective on the thermal stability of composite films.

#### 2.1.4. Thermal Properties Study of Polyimide/SiO_2_ Composite Films

**Figure 4:**
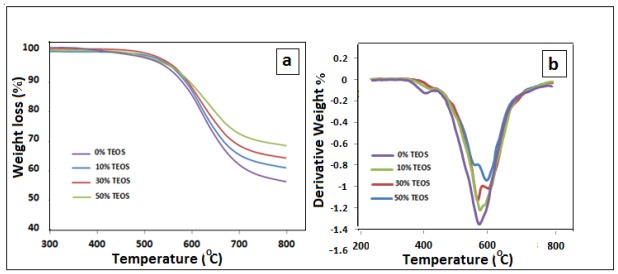
(**a**) TGA and (**b**) DTA thermograms of the polyimide/SiO_2_ composite films with different percentages of TEOS.

The thermal stability of the prepared polyimide/SiO_2_ composite films can be evaluated by TGA. The TG curves of the polyimide/SiO_2_ composite films with various SiO_2_ contents at heating rate of 5 °C/min are shown in Figure 4a. The TG curves indicate that water or solvent has been successfully eliminated from the polyimide film and also polyimide/SiO_2_ composite films because there is no weight loss below 100 °C. It can be clearly seen in Figure 4a that the residual weight of polyimide/SiO_2_ composite films after thermal decomposition is higher than polyimide film above 700 °C. The increase in the weight residues above 700 °C illustrates successful incorporation of higher amounts of silica into the polyimide/SiO_2_ composite films and ultimately increases in thermal stability. The temperatures of the thermal decomposition (*T*_d_) of the polyimide film and polyimide composite films are compared in Table 1. Results show that thermal decomposition of composite films increases with the increase of SiO_2_ contents leading to the assumption that the inorganic components, such as SiO_2_, can improve the thermal stability of organic materials. The improvement of the thermal stability of the prepared polyimide with SiO_2_ can be based on the fact that these materials have inherently good thermal stability and also due to the strong interaction/chemical bonding that exists between the polyimide and silica [16,17].

### 2.2. Degradation Kinetic Analysis

#### 2.2.1. Theoretical Background

One application of the thermogravimetric analysis is the determination of the kinetic parameters, such as reaction order, activation energy, *etc.* In the thermogravimetric analysis, the rate of reaction may be defined as the ratio of the actual weight loss to the total weight loss corresponding to the degradation process [8];

(1) $$X=\frac{\left(W_{0}-W_{\text{t}}\right)}{\left(W_{0}-W_{\text{f}}\right)}$$

where *W*_0_ is the initial weight of the sample, *W*_t_ is the actual weight of the sample, *W*_f_ is the final weight of the sample and *X* is the degree of decomposition.

A typical model for a kinetic process can be represented by the decomposition rate *(dX*/*dt)* which is a function of temperature and weight of the sample. The decomposition rate can be expressed as:

(2) dX/dt=kf(X)

where *dX/dt* is the decomposition rate, *k* is the rate constant and *f*(*X*) is the differential expression of a kinetic model function. However, the rate constant k can be defined by the Arrhenius expression:

(3) k=A exp (-E/RT)

*A* is the pre-exponential factor (s^−1^), E is the activation energy of the degradation reaction (kJ/mol), *R* is the universal gas constant (8.314 J/mol·K) and *T* is the absolute temperature (K). The combination of Equations (2) and (3) leads to the following equation:

(4) dX/dt=A exp (-E/RT)f(X)

In the thermogravimetric analysis, the sample temperature can be changed with a constant heating rate *β (β* = *dT*/*dt*), whereby, whit the introduction of *β*, Equation (4) can be modified as follows:

(5) $$dX/dt=A/\beta \;\text{exp}\;\left(-\frac{E}{RT}\right)f(X)$$

Therefore, Equation (5) is a fundamental relation that determines the kinetic parameters on the basis of thermogravimetric data. Based on the degree of conversion measurement, *X*, and also the heating rate, *β* there are several methods available for the calculation of the apparent activation energy. Hence, the calculation of the kinetic parameters for the degradation from the thermogravimetric analysis data is strongly dependent on the method of calculation. There are a number of methods used to determine the apparent activation energy based on one or different heating rates of the TGA curves and these include Ozawa, Kissinger, van Krevelen, Coatse-Redfern, *etc.* [9]. In the present study, the Ozawa’s method was employed to calculate the apparent activation energy of the thermal degradation of the polyimide (4-APS/BTDA) and polyimide (4-APS/BTDA)/SiO_2_ composite films.

#### 2.2.2. Flynn–Wall–Ozawa Method

The activation energy of the decomposition process can be calculated using the Flynn–Wall–Ozawa’s method without knowing reaction order and differential data of TGA [18,19]. The integration of Equation (5) from an initial temperature T_0_, corresponding to a degree of conversion *X*_0_, to the peak temperature *T*_p_, where *X* = *X*_p_, gives:

(6) $$g(X)=\int_{X_{0}}^{X_{p}}\frac{dX}{f(X)}=\frac{A}{\beta }\int_{T_{0}}^{T_{P}}\;\text{exp }\left(-\frac{E}{RT}\right)dT$$

where *g*(*X*) is the integral function of conversion. Assuming *x* = *E*/*RT*, Equation (6) can be written as:

(7) $$\frac{A}{\beta }\int_{T_{0}}^{T_{P}}\;\text{exp }\left(-\frac{E}{RT}\right)dT=\frac{AE}{\beta R}p(x)$$

Ozawa’s method is based on Doyle’s approximation.

Log *p*(*x*) ≈ 2.315 − 0.457*x*

Or ln*p*(*x*) ≈ 5.330 − 1.052*x*

For 20 < *x* < 60, Equation (7) can be written as:

(8) $$\text{log }\beta =\;\text{log}\frac{AE}{g(X)R}-2.315-\frac{0.4567E}{RT}$$

Here, *A* and *R* are constant and for a particular conversion, *g*(*X*) is a constant. Hence, the value of *E* can be computed by Ozawa’s method for any particular degree of decomposition, being determined from the linear dependence of log *β versus* 1/*T* plot at different heating rates without knowing of the reaction order.

To determine apparent activation energy using Ozawa’s method, several TGA curves at different heating rates (*β*) are essential. Hence, the dynamic thermogravimetric analysis of polyimide and prepared composite films were performed at various heating rates, namely 5, 10, 15 and 20 °C/min in N_2_.

**Figure 5:**
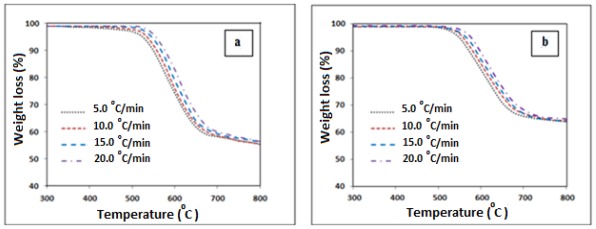
TGA Thermograms of (**a**) the polyimide and (**b**) the polyimide/SiO_2_ composite film prepared with 50% TEOS loading at different heating rates.

Figure 5 shows the thermal degradation curves of the polyimide and polyimide/SiO_2_ composite film with 50% of TEOS loading at different heating rates of 5, 10, 15 and 20 °C/min. As depicted in the figure, the onset decomposition temperature increased with increase of the heating rates for both the compositions. The activation energy of the thermal degradation for pure polyimide and composite films were obtained using the Ozawa’s method, Equation (8), from a linear form a of log *β versus* 1000/*T* at a fixed conversion with the slope of such a line being −0.4567*E*/*RT*.

**Figure 6:**
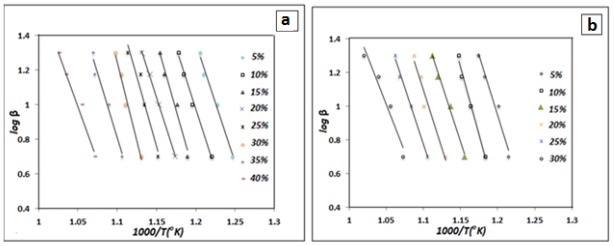
Flynn–Wall–Ozawa plots at varying conversion for the degradation of (**a**) polyimide and (**b**) polyimide/SiO_2_ composite film with 50 wt% TEOS loading.

Figure 6 depicts the relationship between log *β* and 1000/*T* (K) for the different weight loss values. In this study, the chosen conversion values were 5, 10, 15, 20, 25, 30, 35, and 40% for polyimide and 5, 10, 15, 20, 25 and 30 wt% for the polyimide/SiO_2_ composite films during the thermal degradation in nitrogen atmosphere. Similar observations were made for the nanocomposite films polyamide with 10 and 30 wt% TEOS loading according to Figure 6 which are not shown in the figures. The isoconversional plots are parallel straight lines that indicate a complex weight loss process with several mechanisms. The activation energies for every composition can be calculated from the slopes that correspond to the different conversions.

**Figure 7:**
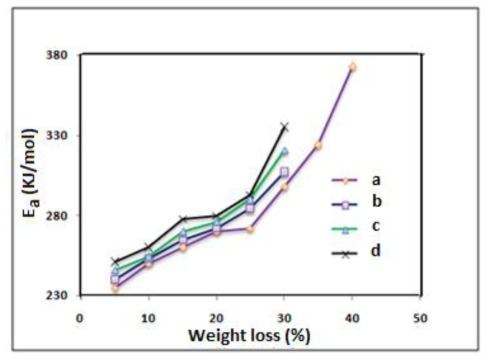
The percentage weight loss dependence of activation energy for the thermal degradation of (**a**) polyimide and (**b**–**d**) polyamide/SiO_2_ composite films with 10, 30, 50 wt% of TEOS loading respectively.

Figure 7 illustrates the values of the activation energies for the thermal degradation of the polyimide and the prepared polyimide/SiO_2_ composite films with different loading of TEOS *versus* percentage of weight loss in the nitrogen atmosphere. As depicted, the values of the activation energies vary with the percentages of weight loss for all compositions. From these curves, the mean activation energies of 258.7, 261.4, 266.5 and 272.4 kJ/mol were calculated for the polyimide pure and its composites with 10, 30 and 50 wt% of TEOS loading, respectively. The activation energies gradually increased with a smooth slope for the pure polyimide before 30% and this was before 25% of weight loss for the polyimide composite films, however, after these values, a jump in the activation energy was observed. This might be due to the residue formed during the thermal degradation. This jump in the polyimide composite films happened sooner; this could be due to the presence of SiO_2_ particles which had been homogeneously dispersed in the polyimide matrix. The dispersed SiO_2_ particles in structure of the prepared polyimide composite films can prevent the permeability of volatile decomposition product from the polyimide.

## 3. Experimental

### 3.1. Materials

4-Aminophenyl sulfone (4-APS) (Aldrich, 97% purity) as diamine and 3,3′,4,4′-benzophenontetra carboxylic dianhydride (BTDA) (Aldrich, 96% purity) as dianhydride were purchased from Sigma-Aldrich, St. Louis, MO, USA, and they were used as the monomers without further purification. *N*-Methyl-2-pyrrolidinone (NMP) (99.5%, extra pure, b.p. 202 °C), which was obtained from Acros Organics, was used as the solvent. Purchased Tetraethyl orthosilicate (TEOS) 98% as SiO_2_ source from Acros Organics was used without further purification.

### 3.2. Preparation of the Polyimide

As a representative procedure, the polyimide can be prepared through thermal imidization. For this purpose a solution of dianhydride monomer BTDA (0.483 g, 1.5 mmol) in NMP (3.0 g) was gradually added to a stirred solution of diamine monomer 4-APS (0.372 g, 1.5 mmol) in NMP (3.0 g) into a 50 mL round-bottomed flask that was equipped with a mechanical stirrer. The mixture was stirred at the room temperature for 24 h to allow viscosity to increase. The prepared PAA solution was subsequently cast onto a clean glass plate. The cast film was dried in an oven at 80 °C for 5 h and then heated at different temperatures and durations (125 °C for 2 h, 150 °C for 2 h, 180 °C for 1 h, 200 °C for 1 h, 250 °C for 1 h and 300 °C for 0.5 h) to convert the PAA into a uniform polyimide film [20] and transparent in yellow color with thickness 90 μm. The temperature and time are both important factors in thermal imidization processing of poly(amic acid) components. In thermal imidization processing the imide ring is formed with elimination of H_2_O molecule from the amic acid and carboxylic acid groups in poly(amic acid) chains. The results showed that the elimination reaction is not relatively fast. Hence, it’s necessary selection of the appropriate combination of temperature and time in the thermal treatment experiments to gradual removal of solvent and formation of imide group rings.

### 3.3. Preparation of the Polyimide/SiO_2_ Composite by Sol-Gel Process

**Figure 8:**
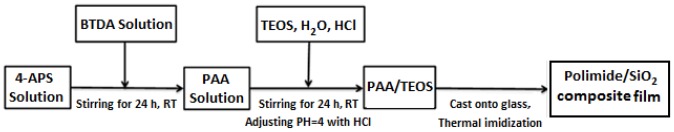
Flow sheet explaining the fabrication of polyimide/SiO_2_ composite film.

Sol-gel process was employed in the synthesis of the polyimide/SiO_2_ composite films, as depicted in Figure 8. 0.372 g 4-APS was added to a round bottom flask and dissolved in NMP by stirring. An equimolar amount of BTDA (0.483 g) solution in NMP was then added to the prepared 4-APS solution. The mixture was stirred continually for 24 h, and finally, a mixture of TEOS and distilled water (4/1 based on TEOS molars) was added into prepared PAA solution 12 wt%. Hydrochloric acid (HCl) was also added to maintain a pH of 4 and then, the mixture was stirred at room temperature for 24 h to yield a transparent solution. The sol-gel process in preparation of PAA was performed in two steps; namely the hydrolysis of alkoxides to produce the hydroxyl group, and residual alkoxides group to form a three-dimensional network [4]. The obtained solution was cast on a clean glass plate and thermally treated in an oven, as explained Section 3.2. The polyimide/SiO_2_ composite films obtained in brownish color with thickness 90 μm. The polyimide/SiO_2_ composite films with various contents of SiO_2_ were prepared according to Table 2.

### 3.4. Characterization

The products of the prepared polyimide and polyimide/SiO_2_ composite films were characterized by FTIR spectra (Perkin-Elmer Model: 100 Series). The created SiO_2_ particles into polyimide matrix were investigated using X-ray diffractometer (Shimadzu, Model XRD 6000). The XRD patterns were recorded at a scan speed of 4 °C/min. The fracture surface morphology of the polyimide nanocomposite films were observed by scanning electron microscopy (SEM) using a LEO 1455 VPSEM. The fracture surfaces were sputter-coated with gold before viewing to eliminate electron charging effect. The particles size distribution was determined using the UTHSCSA image Tool Software (Version 3.00). The thermal properties were determined using the thermogravimetric analysis (Perkin-Elmer, Model TGA-7). Experiments were performed at different heating rate of 5, 10, 15 and 20 °C/min in N_2_. The temperature range for TGA measurements were from 35 to 800 °C.

## 4. Conclusions

Polyimide (4-APS/BTDA)/SiO_2_ composite films with various TEOS loadings were prepared by sol-gel process. Synthesis of polyimide (4-APS/BTDA) and formation of SiO_2_ particles were confirmed by FTIR spectroscopy and X-ray diffraction techniques. The SEM microphotographs of the cross-section surfaces of polyimide/SiO_2_ composite films showed that the created white globular SiO_2_ particles were dispersed evenly in the polyimide matrix. On the basis of morphological observations, the average size of SiO_2_ particles increased with the increase of the TEOS loading. The TG curves of the polyimide/SiO_2_ composite films with various SiO_2_ contents showed that the thermal stability of the prepared polyimide composite films increased with increased SiO_2_ content. The thermogravimetric analysis results from the prepared polyimide composite films also illustrated the apparent activation energies of the thermal decomposition are gradually increased with increased SiO_2_ content. The dispersed SiO_2_ particles, in the structure of the prepared polyimide composite films, may be able to prevent the permeability of volatile decomposition products from the polyimide.

## Figures and Tables

**Table 1:** Comparison of *T*_d_ and average size of SiO_2_ particles in prepared polyimide/SiO_2_ composite films with different percentage of TEOS.

Composite material	TEOS (%)	*T*_d_ (°C)	SiO_2_ average size (nm) ± SD
Sample 1	0	574	-
Sample 2	10	579	264.33 ± 48.44
Sample 3	30	587	373.84 ± 41.60
Sample 4	50	592	579.67 ± 65.37

**Table 2:** The formulations of polyimide/SiO_2_ composite films.

composite material	TEOS (wt%)	PAA (g)	H_2_O (g)	HCl _a_
Sample 1	0	1.7	0	0
Sample 2	10	1.7	0.058	5
Sample 3	30	1.7	0.173	5
Sample 4	50	1.7	0.296	5

a Weight percentage of HCl based on amount of water added.
